# Biosecurity level and health management practices in 60 Swedish farrow-to-finish herds

**DOI:** 10.1186/s13028-015-0103-5

**Published:** 2015-03-12

**Authors:** Annette Backhans, Marie Sjölund, Ann Lindberg, Ulf Emanuelson

**Affiliations:** Department of Clinical Sciences, Swedish University of Agricultural Sciences, SE-750 07 Uppsala, Sweden; Department of Animal Health and Antimicrobial Strategies, National Veterinary Institute, SE-751 89 Uppsala, Sweden; Department of Epidemiology and Disease Control, National Veterinary Institute, SE-751 89 Uppsala, Sweden

**Keywords:** Pig production, Biosecurity, Health management

## Abstract

**Background:**

Biosecurity measures are important tools to maintain animal health in pig herds. Within the MINAPIG project, whose overall aim is to evaluate strategies to raise pigs with less antimicrobial use, biosecurity was evaluated in medium to large farrow-to-finish pig herds in Sweden. In 60 farrow-to-finish herds with more than 100 sows, the biosecurity level was evaluated using a previously developed protocol (BioCheck). In a detailed questionnaire, internal and external biosecurity was scored in six subcategories each. An overall score for biosecurity was also provided. Information regarding production parameters as well as gender and educational level of personnel working with the pigs was also collected. Descriptive statistics were used to examine the recorded data.

**Results:**

The median scores for external and internal biosecurity were 68 and 59, respectively, where 0 indicates total absence of biosecurity and 100 means maximal possible biosecurity. The subcategories for external and internal biosecurity that had the highest scores were “Purchase of animals” (external) and “Nursery unit”/“Fattening unit” (internal), while “Feed, water and equipment supplies” (external) and “Measures between compartments and equipment”/“Cleaning and disinfection” (internal) received the lowest scores. A female caretaker in the farrowing unit, a farmer with fewer years of experience and more educated personnel were positively associated with higher scores for some of the external and internal subcategories. In herds with <190 sows, fattening pigs were mixed between batches significantly more often than in larger herds.

**Conclusions:**

The herds in this study had a high level of external biosecurity, as well as good internal biosecurity. Strong biosecurity related to the purchase of animals, protocols for visitors, the use of all-in, all-out systems, and sanitary period between batches. Still, there is room for improvement in preventing both the introduction of disease to herds (external) and the spread of infections within herds (internal). Systems for animal transport can be improved and with respect to internal biosecurity, there is especially room for improvement regarding hygiene measures in and between compartments, as well as the staff’s working procedures between different groups of pigs.

**Electronic supplementary material:**

The online version of this article (doi:10.1186/s13028-015-0103-5) contains supplementary material, which is available to authorized users.

## Background

In pig herds, biosecurity is an important aspect of preventing the transmission of diseases, thus improving health and reducing the need for antimicrobials (AMs) [1]. External biosecurity aims to keep transmissible pathogens out of the herd, while internal biosecurity prevents the spread of disease, mainly from older to younger animals within the herd [2]. The interest in improving biosecurity in pig production has increased as awareness about the need for AM reduction to prevent the development of resistant bacteria has grown. In order to improve biosecurity, with the aim to minimize AM use in individual herds and at a national level, we need to assess biosecurity in detail, so as to find weaknesses where there is potential for improvement. Several studies in different countries have been published describing biosecurity measures in pig farming [3-9], and one previous study describes biosecurity in Swedish livestock farms including pig herds [10]. To date however, there is no detailed description of how biosecurity is implemented in Swedish pig production.

Sweden, situated in Scandinavia in northern Europe, produces around 2.5 million slaughter pigs yearly, corresponding to 1% of EU production [11]. Most of the industry is concentrated in the south and southwest of the country. Pig production in Sweden has undergone major structural changes during the last decades, resulting in a substantial reduction in the number of holdings with sows and boars while during the same period, herd sizes have increased 3.5 times [12]. In Swedish pig production, the use of antibiotics as growth promoters has been banned since 1986, sows have been kept in loose housing during all production stages including lactation since 1996 [13] and tail docking has never been practised. As these practices apply to pig production in Sweden but not necessarily in other countries, differences in practices imply that results on health and biosecurity measures from other countries are not necessarily applicable to the Swedish situation.

Within MINAPIG (www.minapig.eu), an EU project with the overall aim to evaluate strategies to raise pigs with less AM use, a cross-sectional study was conducted to assess the level of biosecurity in farrow-to-finish herds in four European countries with different levels of AM use. The aim of this study was to describe biosecurity and health management practices in Swedish farrow-to-finish herds using an established scoring system, based on interviews with the farmers.

## Materials and methods

### Selection of participating herds and herd visits

Information on and an invitation to participate in the study was sent out to 100 Swedish farmers with farrow-to-finish herds consisting of at least 100 sows. The source population were either herds affiliated to the Swedish Animal Health Service AB (SvDHV), i.e. herds in which veterinarians from the SvDHV served as herd veterinarians, or herds with previous contact with researchers at the National Veterinary Institute, in Uppsala. In total, a convenience sample of 60 herds was recruited from across Sweden. Recruited herds were visited once during the period April to September 2013, either by the herd veterinarian (48 herds) or by the first or second author (12 herds). Herd veterinarians (n = 15) were instructed on how to perform the visit/interview before the start of the study. The farmer was interviewed about biosecurity measures and a tour around the farm was conducted. An amount of approximately EUR330 was paid in compensation to each participating farm at the end of the study.

### Herd practices related to biosecurity

To evaluate the biosecurity in the herds, a pre-established protocol, BioCheck (available at www.biocheck.ugent.be), was used. BioCheck was originally developed by Laanen *et al.* [1,7] and consists of a total of 109 questions grouped into six subcategories for external and six subcategories for internal biosecurity. Subcategories regarding external biosecurity are: “Purchase of animals and semen”; “Transport of animals and removal of manure and dead animals”; “Feed, water and equipment supplies”; “Personnel and visitors”; “Vermin and bird control”; and “The environment and region”. Subcategories related to internal biosecurity are: “Disease management”; “The farrowing and suckling period”; “The nursery unit”; “The fattening unit”; “Biosecurity measures between compartments and the use of equipment”; and “Cleaning and disinfection”.

Briefly, points were allotted for questions within the subcategories, with each given a weighting factor depending on its estimated importance for the introduction and spread of infectious diseases, as defined by Laanen *et al*. [1,7]. The weights of the questions were subsequently combined into scores for each subcategory, which were further weighted and combined into scores between 0 and 100 for internal and external biosecurity, respectively, where 0 corresponded to “total absence of biosecurity” and 100 to “perfect biosecurity”, i.e. maximal possible biosecurity [1]. Finally, the mean of the scores for external and internal biosecurity was calculated as a whole-herd score.

All questions in the BioCheck form were translated from English into Swedish and questions about production parameters, preventive measures such as vaccination routines, and the educational level, gender and years of experience of the staff member responsible for pig management were also included. The questions were answered by the manager of the pig farm and the interviews were conducted in Swedish. After the visit, the results were manually registered using the online tool, modified for the MINAPIG project.

### Data analysis

The distribution of responses was examined with descriptive statistics. Correlations between total or subcategory scores and herd size, number of weaned piglets per sow per year, or years of pig farming experience was evaluated. Secondly variables were categorized, using medians as the breakpoint, into smaller (range 96–185 sows) and larger herds (190–1200 sows), and farms where farmers had <23 (range 5–22) versus ≥23 (23–41) years of experience, and differences in overall scores and subcategory scores were compared between the groups. Also, differences in overall scores and subcategory scores between farms with female versus male workers responsible for piglet and sow care were studied, between farms with basic – lower versus higher – university education level of responsible person, and between conventional farms and outdoor herds, satellite herds and SPF herds respectively. The significance of the differences between groups was assessed by Mann–Whitney test. Microsoft Excel (Microsoft Corporation, Redmond, WA, USA) and Minitab 16 (Minitab Inc., Harrisburg, PA, USA) were used for data handling and statistical analyses.

## Results and discussion

### Description of the herds, production parameters

Nineteen (32%) of the 60 herds in the study were satellites in a sow pool, a system whereby pregnant sows are leased from a central herd unit to several other herds (“satellite” herds). The sows are transported to the satellite herds before farrowing, and return to the central unit after weaning. Three (5%) were specific pathogen-free (SPF) herds and three (5%) had outdoor production. For Swedish herds altogether, the corresponding figures are 25% sow pool herds, 4% SPF and 1% organic herds, which in Sweden stipulates outdoor production [14,15]. Seventy per cent of the herds reported using a sow management programme. In Table 1, production parameters for the herds in this study are compared with national production data for 2013. The latter were provided by the sow management programme PigWin (www.svenskapig.se) and are based on reports on 59,000 sows (39% of the total Swedish sow population for that year). The average herd size of herds participating in this study was considerably lower than the mean figures from PigWin data, but production numbers, mortality figures and age at weaning were comparable. Our herd size (see Table 1) was, however, approximately the same as the average herd size (190 sows) reported by the Swedish Board of Agriculture [12]. Taken together, the participating herds seemed to represent Swedish pig herds reasonably well regarding production types, herd size and production results.

**Table 1:** **Production data and other parameters (mean PigWin** ^**a**^ **figures) for 60 Swedish farrow-to-finish herds**

**Production data and other parameters**	**Min**	**Median**	**Max**	**Mean**	**SD**	**Mean PigWin, 2013**
**Sows (n)**	96.0	188	1200	243	179	309
**Litters/sow/year**	1.60	2.20	2.40	2.20	0.14	2.21
**Weaned piglets/sow/year**	14.1	23.6	28.3	23.2	2.3	24.0
**Mortality till weaning (%)**	2.3	17.8	36.5	17.9	0.05	17.9
**Age at weaning (days)**	28.0	35.0	49.0	35.1	3.7	33.1
**Time in battery (weeks)**	4.0	7.0	12.0	6.9	1.5	6.7
**Time in fattener unit (weeks)**	12	15	19	14.8	1.4	n.s.
**Daily weight gain** ^**b**^ **(g/day)**	800	901	1007	912	57.1	913

n.s. = not specified; SD = standard deviation.

^a^Figures are based on 2013 data from www.pigwin.se/medeltal-sugg,/medeltal-slakt, accessed on 14 October 2014. ^b^Daily weight gain was specified for 38 herds only.

### Information on the pig farm manager and other staff

On the farms included, the person who made biosecurity decisions and who was mainly responsible for the pigs (also referred to as “pig farm manager”) had a median of 23 years (range 5–41 years) of relevant experience in pig farming. The educational level of the pig farm managers was lower agricultural education 38%, university education 27%, higher agricultural education 17%, not specified 13% and basic school education 5%. The gender distribution was 48% female and 52% male in the farrowing and nursing units, and 30% female, 67% male and 3% not stated in the finishing units. The results suggest that the educational level of the farming personnel is appropriate for the task on the majority of farms and that there is a gender balance, at least among persons in charge of pigs, on Swedish pig farms. A median number of three persons (range two to 15) worked regularly in the pig barns.

### Biosecurity level

#### Overall scores and subcategory scores

**Figure 1:**
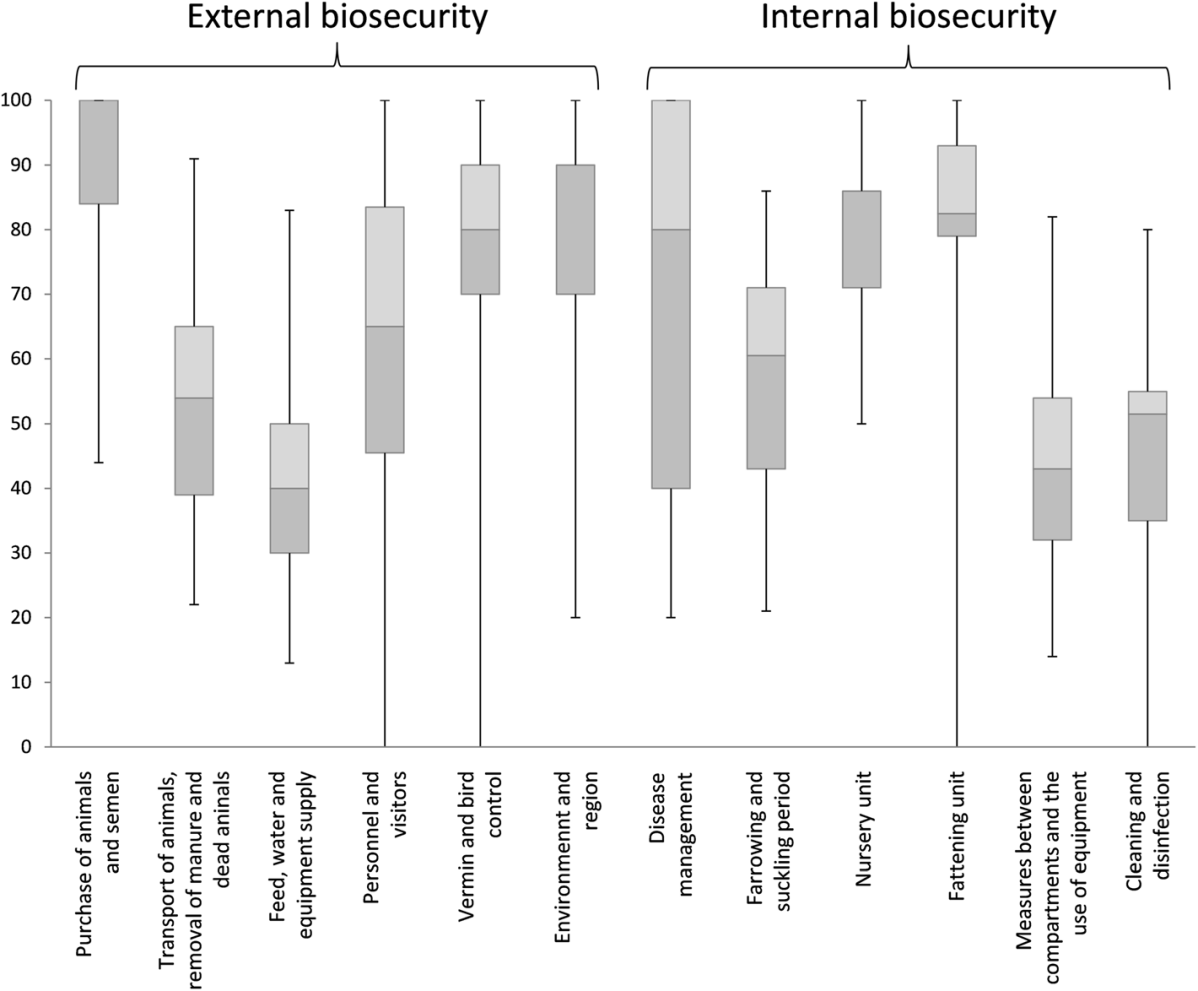
**Box-and-whisker plot of scores for external and internal biosecurity subcategories for 60 Swedish farrow-to-finish herds.** The boxes show first quartiles (light grey), median, third quartiles (dark grey); whiskers show highest and lowest scores.

The average score for external biosecurity was 68 (median 68) and for internal 59 (median 61), while the average total score was 64 (median 65). Distributions of the scores for each subcategory are shown in Figure 1. The external biosecurity subcategory that received the highest score was “Purchase of animals and semen”, while the subcategory with the lowest score was “Feed, water and equipment supplies”. For internal biosecurity, the subcategories “Nursery unit” and “Fattening unit” scored the highest mean scores, while “Biosecurity measures between compartments and the use of equipment” and “Cleaning and disinfection” scored the lowest. A detailed description of separate questions is given in the next section.

The average score for internal biosecurity in the present study was slightly higher compared with Belgian herds (average 52, median 53) described in a recent study by Laanen *et al.* [1] using the same scoring system, while our average scores for external biosecurity were similar to the Belgian scores (65, median 66). The allocation of scores for the different subcategories was similar, except for “The environment and region” where our population scored higher in relation to other subcategories compared with the study by Laanen *et al*. [1]. This is not surprising considering how the two countries differ with regard to pig density.

**Figure 2:**
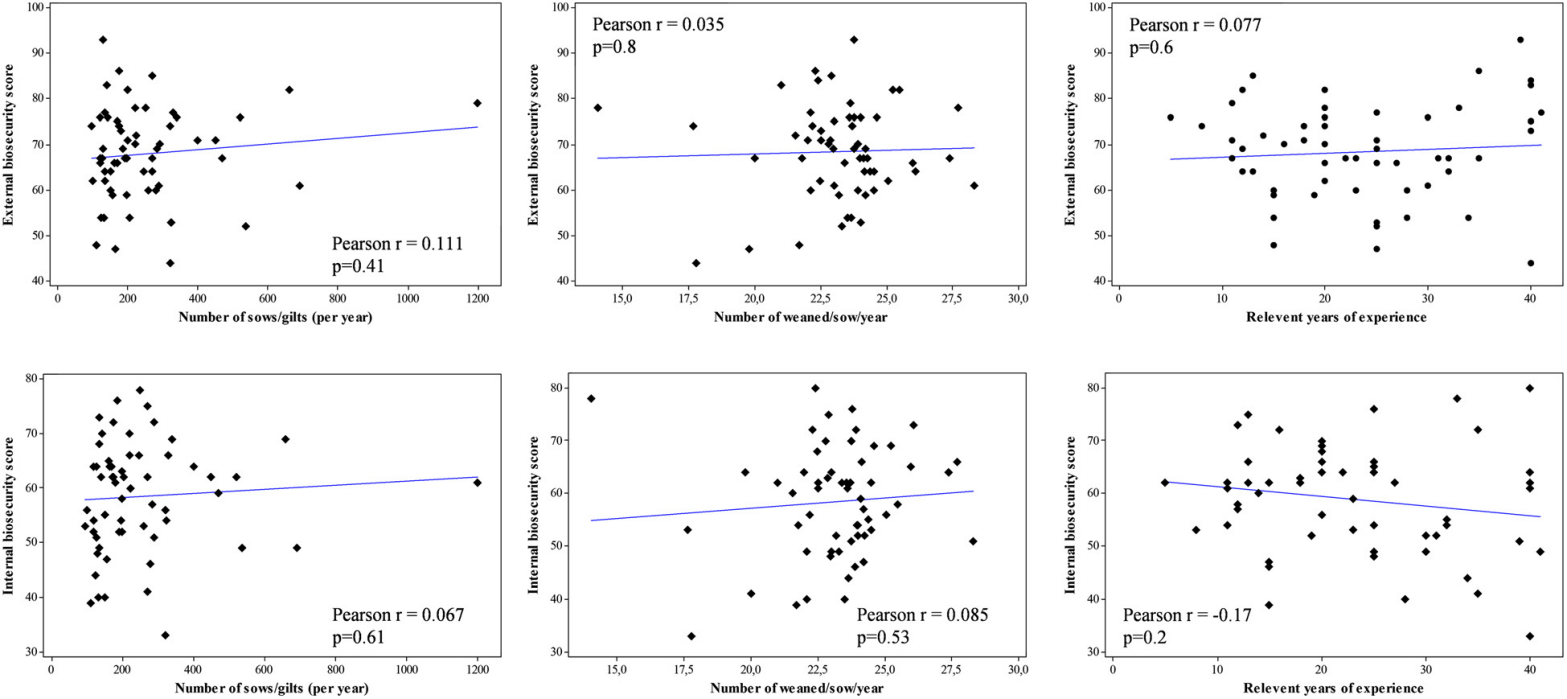
**Correlation between internal and external biosecurity scores and herd size, number of weaned piglets per sow per year, and relevant years of experience for pig farm manager.**

There were no significant correlations between total or subcategory scores and herd size, number of weaned piglets per sow per year, or years of pig farming experience (Figure 2). Larger herds did not score significantly better for any subcategory in contrast to the Belgian results where larger herds scored significantly higher on external biosecurity [1]. However, pig herds are generally larger in Belgium than in Sweden. A closer look at the results showed that one aspect that differed between larger and smaller herds was the mixing of fattening pigs between batches which occurred significantly more often in herds with 96–185 sows than in herds with 190–1200 sows (*P* < 0.01; data not shown). In Table 2, total and subcategory scores between herds with different characteristics are compared. The total score for biosecurity, as well as the subtotal external score, was significantly higher in herds where the staff member responsible for piglets and sows was female. The subcategories that differed significantly between the gender groups were “Disease management”, “Biosecurity measures between compartments and the use of equipment” and “Vermin and bird control”, where herds managed by a female farm worker had higher scores. Likewise, herds where farmers had <23 years of experience scored significantly better on the latter two subcategories The reasons for these differences cannot be concluded from this material and should be subject to further study. The underlying reasons for the gender differences observed cannot be determined from our data, but similar differences have been observed by others and may apply to various extent also to our context. For example, a generally higher empathy towards animals has previously been shown in females [16]; a characteristic which could influence the higher scores seen for disease management. Female farmers have also been shown to have a higher perceived disease knowledge level and to be more confident that they can influence if infections are introduced or not [17]. These differences are also reflected in studies showing how females tend to have higher medical compliance, a higher degree of preventive health behavior and beliefs that indicate a lower level of health risk-taking [18]. This may reflect differences in attitudes that could also affect the extended health behavior directed towards animals.

**Table 2:** **Difference in biosecurity scores according to herd characteristics**

	**Gender of person responsible for biosecurity in farrowing unit: female (upper row) or male (lower row)**			**Herd size: smaller (upper row) or larger (lower row)**			**Years of experience: less (upper row) or more (lower row) than 23 years**			**Conventional herd (upper row) or outdoor production (lower row)**			**Conventional herd (upper row) or satellite herd (lower row)**			**Conventional herd (upper row) or SPF herd (lower row)**			**Responsible person education level basic – lower (upper row) or higher – university (lower row)**		
**Biosecurity category**	**M**	**IQR**	**P-value**	**M**	**IQR**	**P-value**	**M**	**IQR**	**P-value**	**M**	**IQR**	**P-value**	**M**	**IQR**	**P-value**	**M**	**IQR**	**P-value**	**M**	**IQR**	**P-value**
**Purchase of animals and semen**	100	18	0.93	100	17	0.79	100	18	0.8	100	18	0.67	100	18	0.36	100	18	a	100	20	0.47
	100	17		100	15		100	15	7	88	20		100	16		100	0		100	15	
**Transport of animals, removal of manure and dead animals**	62	30	0.09	52	28	0.45	54	25	0.89	55	25	0.58	56	25	0.32	56	25	0.03	58	30	0.47
	50	23		59	27		54	28		50	42		50	31		78	26		53	25	
**Feed, water and equipment supply**	40	20	0.22	40	15	0.34	40	20	0.39	40	20	0.78	40	20	0.55	40	20	0.03	40	20	0.86
	40	20		40	20		40	20		30	27		40	10		73	40		40	20	
**Personnel and visitors**	74	40	0.10	62	49	0.25	71	43	0.56	68	41	0.93	68	41	0.14	68	41	0.01	59	38	0.25
	59	41		71	41		65	50		76	94		59	40		100	6		71	**46**	
**Vermin and bird control**	90	18	**0.02**	80	30	0.11	90	13	**0.01**	80	30	0.64	80	30	0.06	80	30	a	80	**20**	0.89
	80	30		90	30		80	30		70	10		90	18		90	0		80	23	
**Environment and region**	90	20	0.82	90	23	0.63	90	30	0.8	90	20	0.91	90	20	0.18	90	20	a	85	30	0.08
	90	30		90	22		90	20	6	90	80		90	28		90	0		90	30	
**Subtotal external biosecurity score**	71	10	**0.01**	67	14	0.21	70	13	0.5	68	14	0.82	68	14	0.75	68	14	**0.00**	67	14	0.35
	64	14		71	14		67	17	8	70	27		65	14		86	10	**6**	70	13	
**Disease management**	80	40	**0.03**	60	60	0.25	80	45	0.4	60	60	0.20	60	60	0.59	60	60	0.20	60	40	0.29
	40	60		80	60		80	60	2	100	40		80	50		100	40		80	60	
**Farrowing and suckling period**	54	33	0.41	61	23	0.64	54	30	0.8	64	21	0.18	64	21	0.05	64	21	0.59	57	23	0.64
	64	21		61	36		64	28	5	79	15		47	26		64	28		64	36	
**Nursery unit**	86	20	0.59	86	15	0.86	86	15	0.3	86	15	0.21	86	15	0.98	86	15	0.65	71	19	**0.02**
	86	15		86	15		86	15	6	93	*		86	15		71	15		86	11	
**Fattening unit**	79	14	0.13	79	24	0.40	86	14	0.35	79	31	0.09	79	31	0.08	79	31	0.18	86	31	0.64
	93	14			90	14		79	29		93	14	93	14		93	21		83	14	
**Measures between compartments and the use of equipment**	54	22	**0.002**	39	20	0.24	54	19	**0.01**	38	22	0.98	38	22	**0.03**	38	22	0.53	43	22	0.36
	39	21		50	14		39	17		39	25		52	14		47b	np		45	26	
**Cleaning and disinfection**	50	24	0.81	55	29	0.55	55	21	0.1	45	36	1.0	45	36	0.09	45	36	0.69	37	35	**0.03**
	48	20		47	23		45	20	0	45	7		55	17		55	20		55	16	
**Subtotal internal biosecurity score**	62	15	0.09	58	14	0.31	62	12	0.0	55	16	0.28	55	16	**0.05**	55	16	0.44	55	14	**0.03**
	55	13		62	20		54	15	7	64	19		62	10		62	21		62	12	
**Total score**	68	11	**0.01**	64	12	0.25	66	8	0.1	64	14	0.80	64	14	0.36	64	14	**0.03**	63	14	0.12
	63	10		67	14		63	17	8	64	15		65	11		73	7		66	13	

Overall scores and subcategory scores between herds with a female versus male caretaker of piglets and sows (female n = 28, male = 31), smaller (96–185 sows, n = 30) and larger herds (190–1200 sows, n = 29), farmers with <23 years (range 5–22 years, n = 30) of experience and farmers with ≥23 years (range 23–41 years, n = 29) of experience, conventional herds (n = 34) and outdoor production (n = 3), satellite herds (n = 19) and SPF herds (n = 3) and lower (n = 26) and higher education level (n = 30) for personnel among 60 Swedish farrow-to-finish herds. The significance of differences between groups was assessed by Mann–Whitney test. Significant *P*-values are indicated by bold characters.

M = medians, IQR = Interquartile range, a = statistical analysis not possible due to data missing, b = n is 2, np = not possible to calculate IQR.

There were no significant differences in internal, external or total biosecurity scores when comparing conventional farms with farms with outdoor production, but only a few outdoor herds participated. Satellite herds had borderline significantly higher internal biosecurity scores than conventional farms. Not surprisingly, SPF herds had significantly higher score for external biosecurity than conventional farms but not for internal biosecurity. A higher education level for the person responsible for the animals was associated with a significantly higher internal biosecurity score, and the subcategories that were significantly different was the “Nursery period” and “Cleaning and disinfection”.

### Detailed description of external biosecurity

Of 19 herds that purchased breeding pigs, 17 used a quarantine stable where a strict all-in, all-out routine was applied in all cases. The mean minimal length of the quarantine period was 23 days. It should be noted that the satellite herds (n = 19) receive sows that could have spent the previous farrowing period in another satellite herd, without quarantine. However, sows from different satellites will spend the dry period together in the central unit. Therefore, sow pools are considered to be one single epidemiological unit. Only five herds purchased piglets, so these were not investigated further. Forty-two of the herds purchased semen (19 herds were satellite herds to a sow pool, in which the central unit purchases semen; however, two of the satellite herds reported own purchase of semen), but of these, 100% acquired semen from boar stations with a higher or equal health status. For details see Additional file 1. The overall low density of pig farms in Sweden explains the high scores for the subcategory “The environment and region” (median 90), but as many as 65% of herds answered that wild boars had been spotted within 10 km of their farm. Swedish farmers seem to be aware of the risks that rodents, birds and companion animals in the stable can pose. Only one of the herds did not have a rodent control programme and 75% had placed grids in front of air intakes to stop wild birds from entering their stables (“Vermin and bird control”, median 80). This could be compared with a Spanish study where only 54.7% of pig breeding farms applied control programmes for rodents [4]. See Additional file 2 for details.

**Figure 3:**
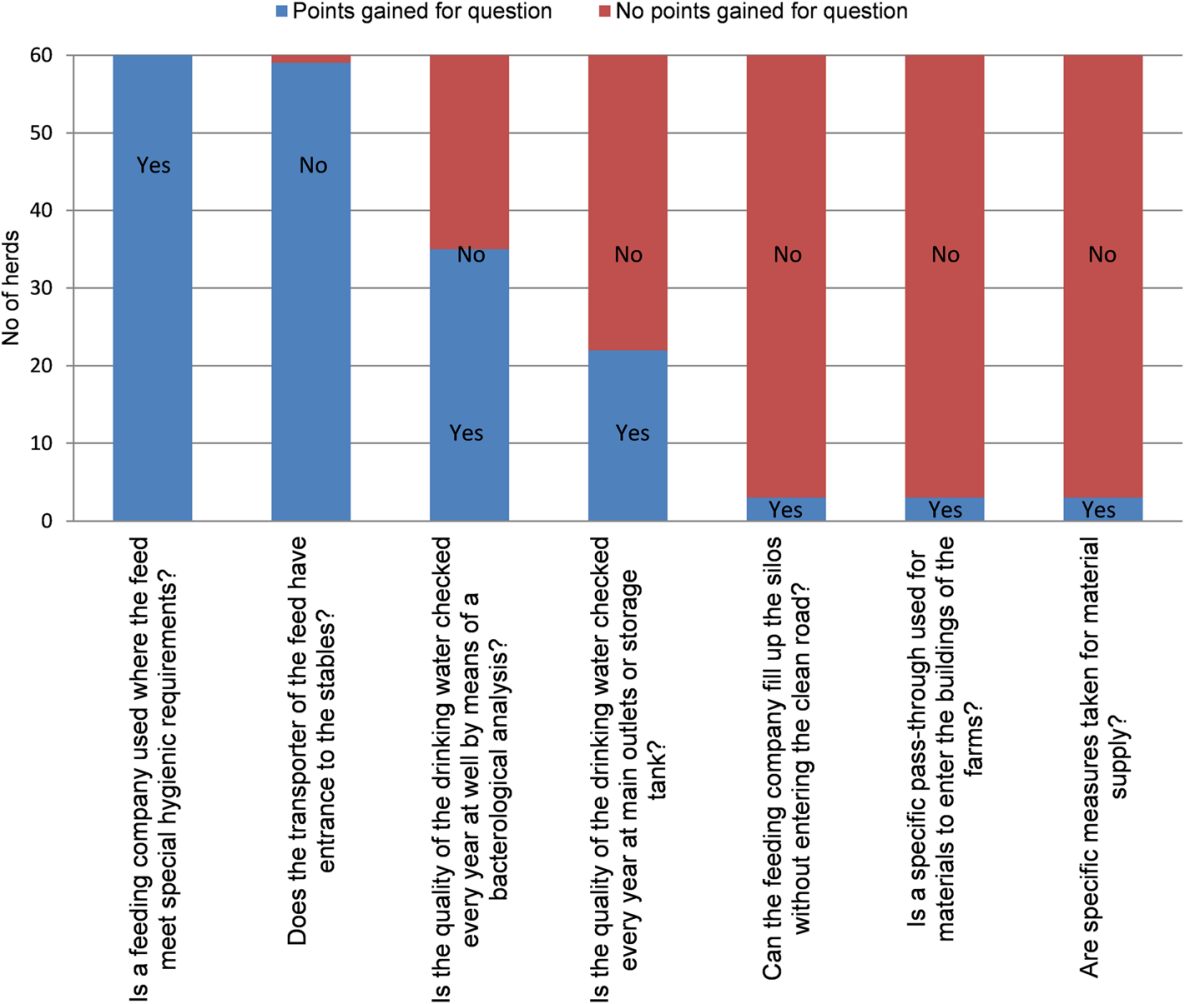
**Answers to questions regarding the subcategory “Feed, water and equipment supplies”.**

**Figure 4:**
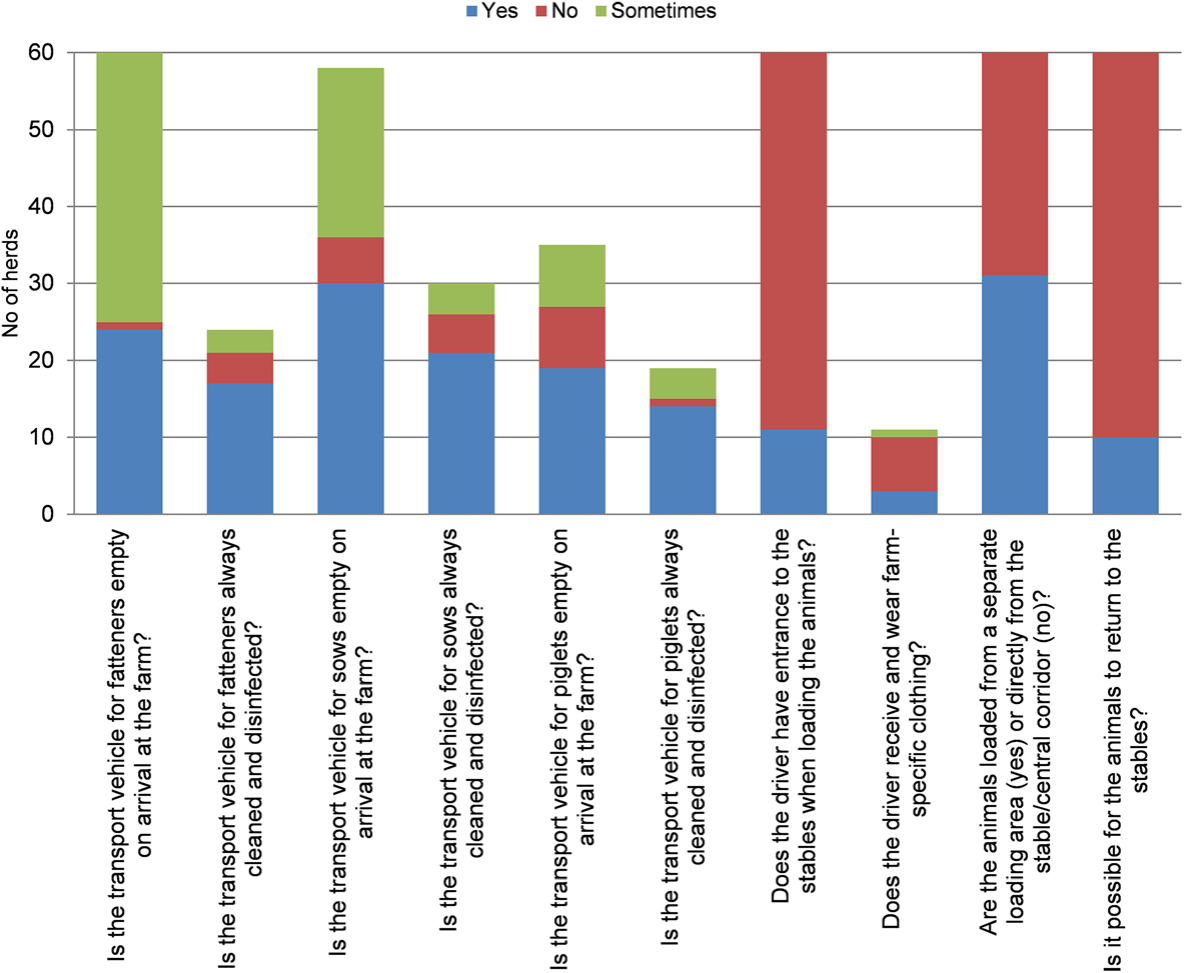
**Answers to questions regarding the subcategory “Transport of animals, and removal of manure and dead animals”.** The low bars indicate that the question was not answered by all the herds; it was skipped when not relevant according to the answer to previous question.

A weak point with the farms included in this study was that 95% of farms answered that feed transports used the clean road, which can be interpreted as a general lack of distinction between clean and dirty roads on Swedish farms. Also, 95% of herds did not have specific measures for pass-through of material supplies (Figure 3). Moreover, about 50% of the farms tested their water quality only once per year. In Sweden, water is generally of good quality but for those herds that have a well of their own, more frequent testing should be recommended. The subcategory “Transport of animals, removal of manure and dead animals” received a median score of 54 (Figure 4). At the majority of farms (83%), it was not possible for the pigs to return to the stables after being in the transport vehicle, nor was the driver able to enter to the stables while loading animals (82%). However, the transport was not empty at arrival for transport of sows in 50% of herds and for fattening pigs in 60% of herds. This could be problematic considering that a separate loading area was only available at 50% of the farms.

The subcategory “Personnel and visitors” received a median score of 65. About 90% of herds provided visitors with boots and clothing, and about 85% obliged visitors to check in before entering the farm. A hygiene lock was used, washing and disinfection of hands was done and a quarantine period of more than 12 hours after visiting other farms was applied for visitors in more than half of the farms. However, these measures were carried out by farmers and staff themselves on only 32% of farms, which reduced the score for this subcategory. Further details are presented in Additional file 3. To summarize, the results of this study show that improvements in external biosecurity can be made regarding transport of animals and feed, e.g. by arranging a separate loading area and implementing a stricter policy for farmer and staff entering the farm.

### Detailed description of internal biosecurity

**Figure 5:**
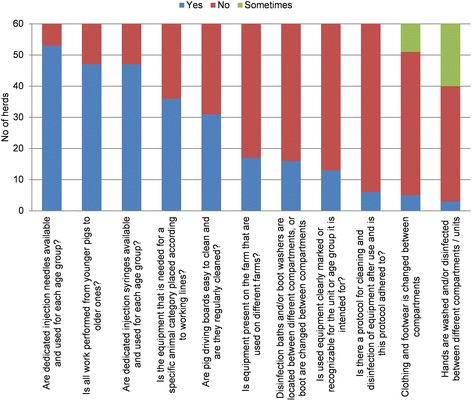
**Answers to questions regarding the subcategory “Measurements between compartments and the use of equipment”.**

An all-in, all-out system in the fattening unit was practised in 90% of herds, for all compartments. In the nursery unit, a strict all-in, all-out protocol was practised at a pen level in 95% of the herds, but the situation at the compartment level was not covered by the questionnaire. In the nursery, 77% of herds achieved the maximum score for a pig density of three or fewer pigs per m^2^, and in the fattening unit 80% of herds had >0.6 m^2^ per pig, which is larger than the space requirement stipulated by the EU (Council Directive 2008/120/EC) [13]. See Additional files 4, 5 and 6 for details. What contributed to the overall low score for the subcategory “Biosecurity measures between compartments” (Figure 5) was that only 8% of farms required their staff to always change clothing and footwear and only 5% got them to always wash their hands between compartments. “Cleaning and disinfection” scored overall low scores because of the limited use of foot baths (5%), a measure that was given considerable weight in this subcategory. It could be argued that measures such as cleaning and disinfection of stables and compartments, implemented by 78%, and a mean sanitary period of 5.33 days between batches, in 92% of herds, are at least as important for biosecurity as are foot baths and that the poor score for the subcategory is not of major concern. See Additional file 7 for more details.

**Figure 6:**
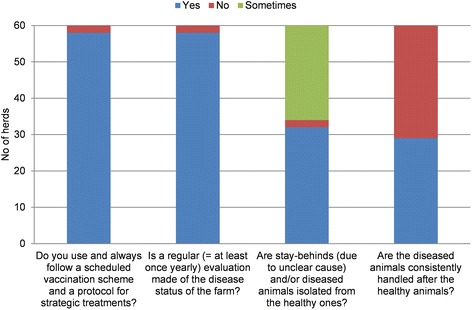
**Answers to questions regarding the internal biosecurity subcategory “Disease management”.**

Scores for “Disease management” varied the most between herds, but were based on four questions only. Strengths within the subcategory were that 97% of the herds had a plan for vaccination and treatments that was complied with, and that the disease status of the herd was regularly checked (Figure 6). Weaknesses were that only half of the herds performed isolation of runts and sick animals and consistently handled sick animals only after handling healthy ones. Based on the results above, improvements in internal biosecurity should focus on establishing stricter hygiene measures for personnel, between compartments.

To the authors’ knowledge, only one previous study, conducted in 2006, describes biosecurity measures in Swedish pig herds, although that also included specialized fattening farms [10]. The study protocols were completely different so comparisons between the 2006 and the present study are difficult to make. Nevertheless, some biosecurity measures were found to be better in the current study than in the previous one. For example, transporters were allowed to enter the stables in fewer herds in our study (28% compared with 44% in the previous study), and there was more use of quarantine for purchased animals (89% versus 74%) and of providing farm-specific clothes for visitors (90% versus 85%) and less sharing of equipment with other farms (28% versus 57%). Part of the difference may be attributable to improvements that have evolved over time, but sampling differences may also be part of the explanation. Also, many pig herds have closed down since 2006 as a result of the difficult economic situation facing the pig industry, and it cannot be excluded that the herds that have closed down were to a greater extent herds with old facilities, perhaps with a reduced possibility for good biosecurity practices.

### Limitations of the study

The source population were mainly herds affiliated to the SvDHV’s full service programme, which excludes herds with herd veterinarians outside this organization. To be able to make cross-country comparisons within the MINAPIG project, the inclusion criterion for herd size was set to >100 sows. In Sweden, more than half of farrow-to-finish herds are smaller [12], and therefore the results should be viewed as representative only of medium-sized and large herds. Furthermore, it cannot be excluded that there was an overrepresentation of farmers with a general interest in questions regarding AM use among those who agreed to participate, considering the overall aim of the study, as farms with a low level of biosecurity or a high level of AM use may have been more reluctant to join. Furthermore, it can be discussed whether the weights given to different factors in BioCheck are entirely relevant and applicable under Swedish conditions since the tool was developed in Belgium, which differs from Sweden regarding infectious diseases in pigs. However, the advantage of using this tool is that it allows for detailed comparisons between herds and also between countries, which is the overall aim of MINAPIG.

## Conclusions

It can be concluded that middle-sized to large Swedish farrow-to-finish systems have a high level of external biosecurity, and a good internal biosecurity. Good routines for purchase of animals and protocols for visitors are applied in the majority of herds. Furthermore, there are low stocking densities and an all-in, all-out protocol is applied in most herds. The external biosecurity can be improved regarding transports for animals and feed. For internal biosecurity, there is room for improvement especially regarding hygiene measures such as washing of hands and changing of clothes and boots in and between compartments and the order in which work is carried out between different groups of pigs. A female caretaker in the nursery unit or a farmer with <23 years of experience was positively associated with higher scores for certain external and internal biosecurity scores, and a higher level of education was likewise associated with higher scores for internal biosecurity.

## Additional files

Additional file 1:
**Answers by 60 Swedish farrow-to-finish herds to questions regarding the external biosecurity subcategory “Purchase of animals and semen”.** The low bars indicate that the question was not answered by all the herds; it was skipped when not relevant according to the answer to previous question. Additional file 2:
**Answers by 60 Swedish farrow-to-finish herds to questions regarding the external biosecurity subcategory “Environment and region”.**
Additional file 3:
**Answers by 60 Swedish farrow-to-finish herds to questions regarding the external biosecurity subcategory “Personnel and visitors”.**
Additional file 4:
**Answers by 60 Swedish farrow-to-finish herds to questions regarding the internal biosecurity subcategory “Farrowing and suckling period”.**
Additional file 5:
**Answers by 57 Swedish farrow-to-finish herds to questions regarding the internal biosecurity subcategory “Nursery unit”.** Three of the 60 herds did not have a separate nursery unit. Additional file 6:
**Answers by 60 Swedish farrow-to-finish herds to questions regarding the internal biosecurity subcategory “Fattening unit”.**
Additional file 7:
**Answers by 60 Swedish farrow-to-finish herds to questions regarding the internal biosecurity subcategory “Cleaning and disinfection”.**

## Abbreviations

AM: Antimicrobials
EU: European Union
SPF: Specific pathogen-free, i.e. a herd free of infection with swine influenza virus, *Actinobacillus pleuropneumoniae*, *Mycoplasma hyopneumoniae*, *Sarcoptes scabiei*, *Brachyspira hyodysenteriae*, toxin-producing *Pasteurella multocida*
SvDHV: Swedish Animal Health Service

## References

[1] Laanen M, Persoons D, Ribbens S, de Jong E, Callens B, Strubbe M (2013). Relationship between biosecurity and production/antimicrobial treatment characteristics in pig herds. Vet J.

[2] Food and Agriculture Organization of the United Nations/World Organisation for Animal Health/World Bank (2010). Good practices for biosecurity in the pig sector – Issues and options in developing and transition countries.

[3] Alawneh JI, Barnes TS, Parke C, Lapuz E, David E, Basinang V (2014). Description of the pig production systems, biosecurity practices and herd health providers in two provinces with high swine density in the Philippines. Prev Vet Med.

[4] Casal J, De Manuel A, Mateu E, Martín M (2007). Biosecurity measures on swine farms in Spain: Perceptions by farmers and their relationship to current on-farm measures. Prev Vet Med.

[5] Simon-Grifé M, Martín-Valls GE, Vilar MJ, García-Bocanegra I, Martín M, Mateu E (2013). Biosecurity practices in Spanish pig herds: Perceptions of farmers and veterinarians of the most important biosecurity measures. Prev Vet Med.

[6] Julio Pinto C, Santiago Urcelay V (2003). Biosecurity practices on intensive pig production systems in Chile. Prev Vet Med.

[7] Laanen M, Beek J, Ribbens S, Vangroenweghe F, Maes D, Dewulf J (2010). Biosecurity on pig herds: Development of an on-line scoring system and the results of the first 99 participating herds. Vlaams Diergen Tijds.

[8] Sahlström L, Virtanen T, Kyyrö J, Lyytikäinen T (2014). Biosecurity on Finnish cattle, pig and sheep farms -results from a questionnaire. Prev Vet Med.

[9] Boklund A, Alban L, Mortensen S, Houe H (2004). Biosecurity in 116 Danish fattening swineherds: descriptive results and factor analysis. Prev Vet Med.

[10] Nöremark M, Frössling J, Lewerin SS (2010). Application of routines that contribute to on-farm biosecurity as reported by Swedish livestock farmers. Transbound Emerg Dis.

[11] Marquer P. Pig farming in the EU, a changing sector. In Eurostat Statistics in focus. vol. 8;2010.

[12] Swedish Board of Agriculture. Livestock in June 2013. Final Statistics, vol. JO20SM 1401. SCB; 2014. http://www.scb.se/en_/Finding-statistics/Publishing-calendar/Show-detailedinformation/?publobjid=23598+

[13] SFS 1988, Swedish Code of statues 1988. 539 Animal Protection Ordinance (in Swedish).

[14] Wallenbeck A. Pigs for organic production. Studies of sow behaviour, piglet-production and GxE interactions for performance. PhD thesis. Swedish University of Agricultural Sciences, Faculty of Veterinary Medicine and Animal Science, Department of Animal Breeding and Genetics; 2009.

[15] Sjölund M. Actinobacillus pleuropneumoniae - A major respiratory pathogen in pigs. PhD thesis. Swedish University of Agricultural Sciences, Faculty of Veterinary Medicine and Animal Science, Department of Clinical Sciences; 2011.

[16] Eckardt Erlanger A, Tsytsarev S (2012). The relationship between empathy and personality in undergraduate students’ attitudes toward nonhuman animals. Society & Animals.

[17] Frössling J, Sternberg-Lewerin S, Nöremark M. Livestock farmers’ perception and opinions on biosecurity. In Proceedings of the Annual Conference of the Society for Veterinary Epidemiology and Preventive Medicine (SVEPM). Dublin; 2014.

[18] Courtenay W, Mccreary D, Merighi J (2002). Gender and ethnic differences in health beliefs and behaviors. J Health Psychol.

