# Supplementary figures and images for: Biosecurity level and health management practices in 60 Swedish farrow-to-finish herds
Source: Acta Vet Scand. 2015 Mar 12;57(1):14. doi: 10.1186/s13028-015-0103-5 (PMC4359795; doi:10.1186/s13028-015-0103-5)

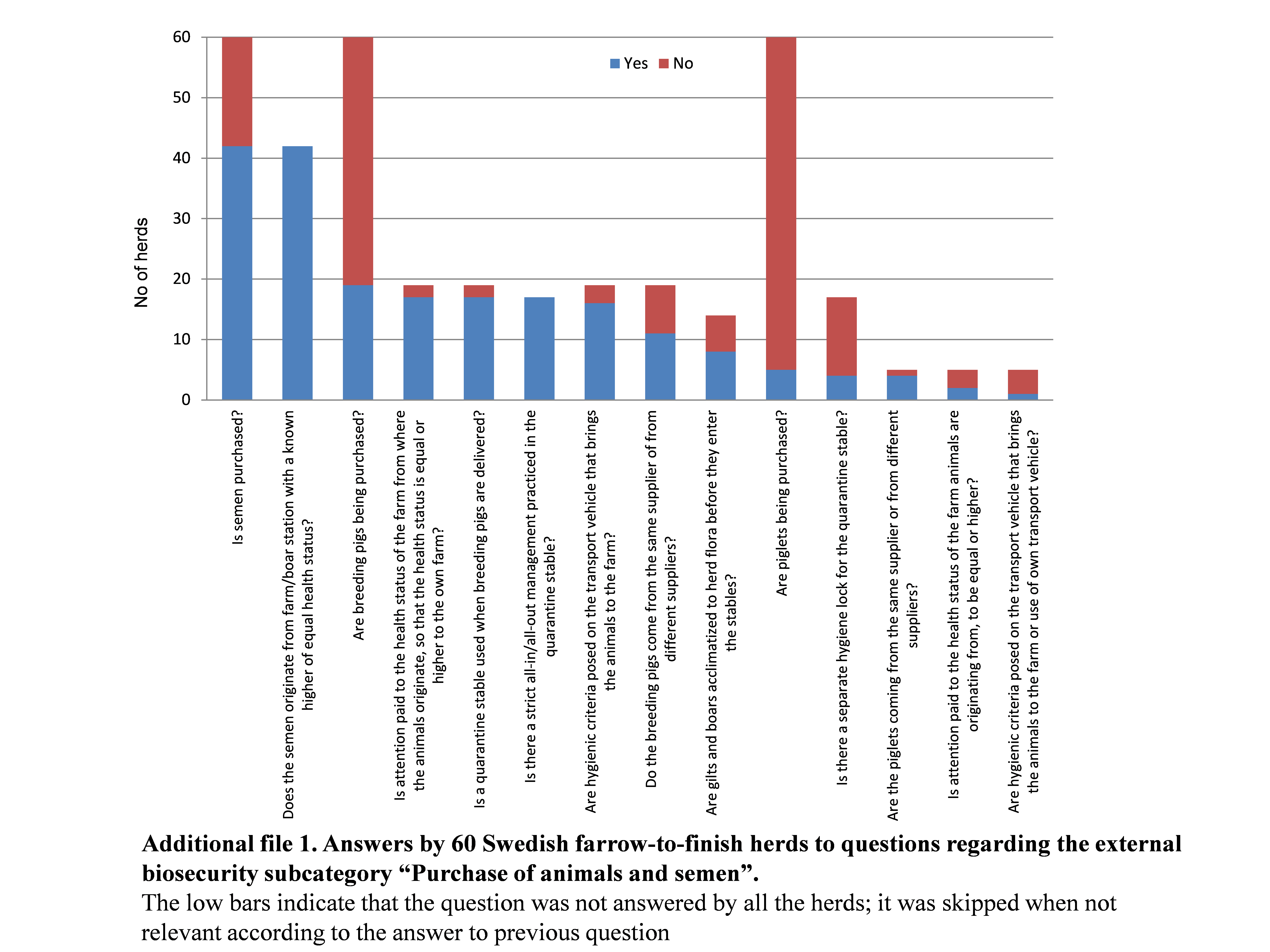

Supplement: Additional file 1: — Answers by 60 Swedish farrow-to-finish herds to questions regarding the external biosecurity subcategory “Purchase of animals and semen”. The low bars indicate that the question was not answered by all the herds; it was skipped when not relevant according to the answer to previous question. [file 13028_2015_103_MOESM1_ESM.png]

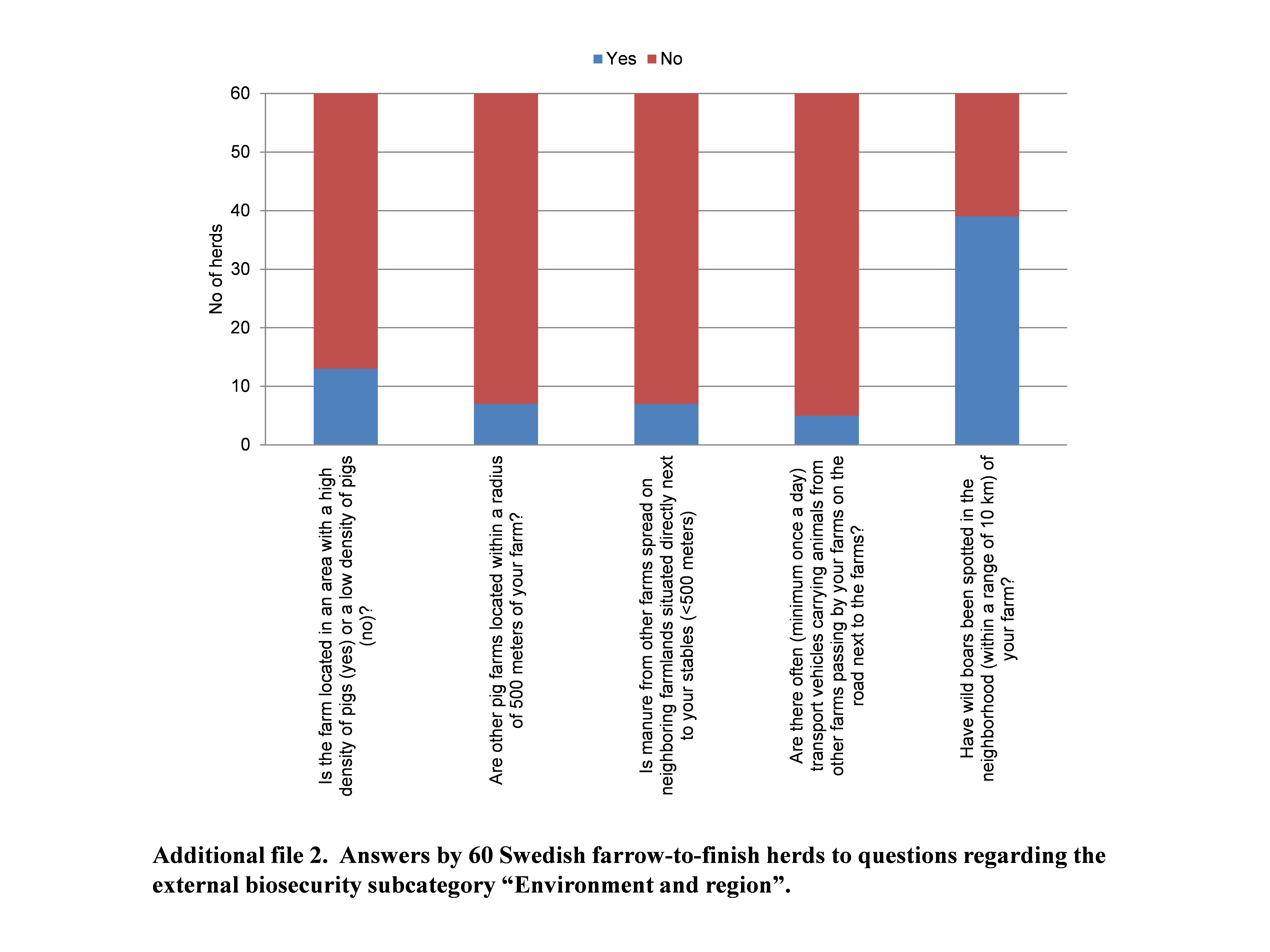

Supplement: Additional file 2: — Answers by 60 Swedish farrow-to-finish herds to questions regarding the external biosecurity subcategory “Environment and region”. [file 13028_2015_103_MOESM2_ESM.png]

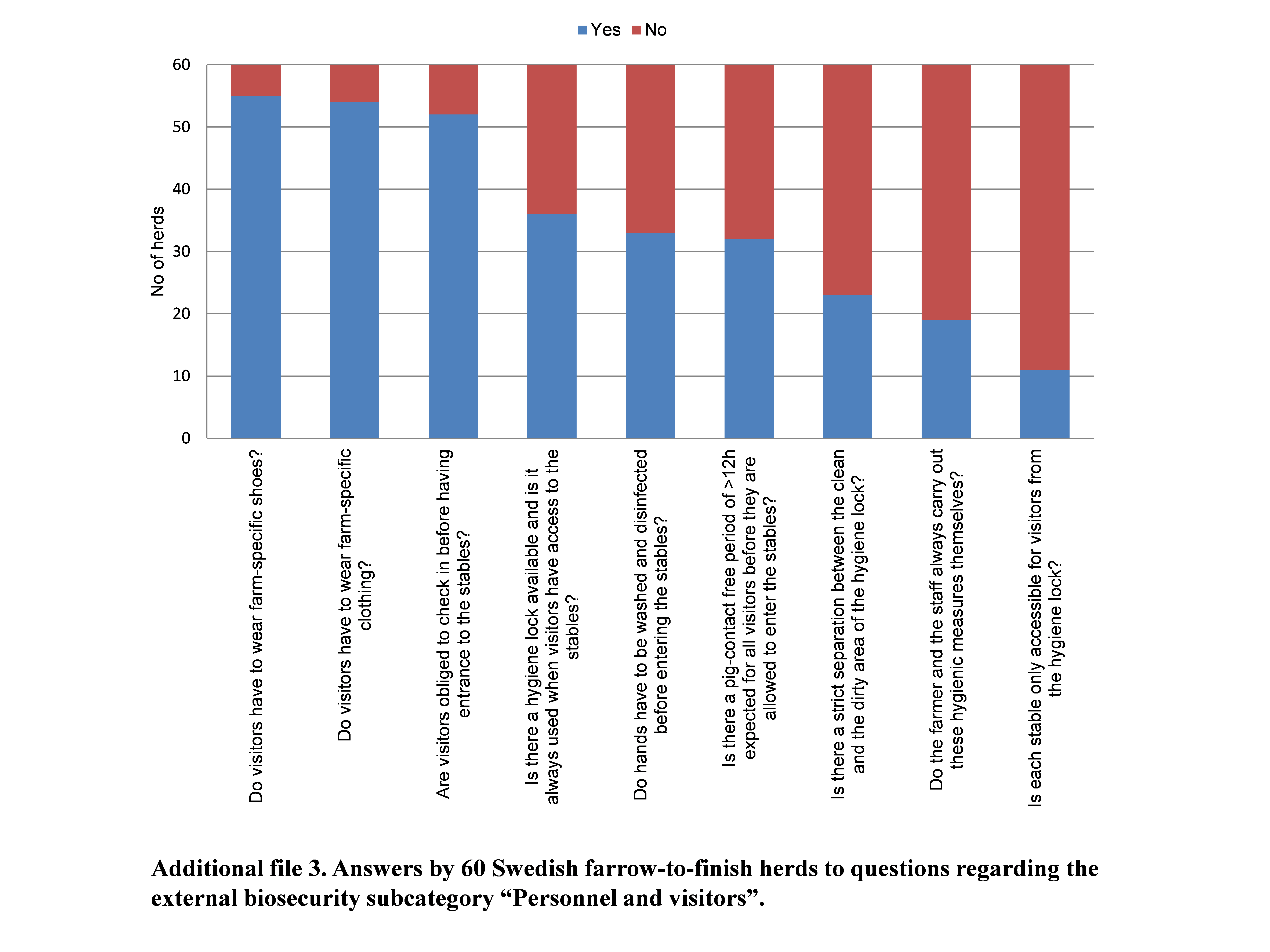

Supplement: Additional file 3: — Answers by 60 Swedish farrow-to-finish herds to questions regarding the external biosecurity subcategory “Personnel and visitors”. [file 13028_2015_103_MOESM3_ESM.png]

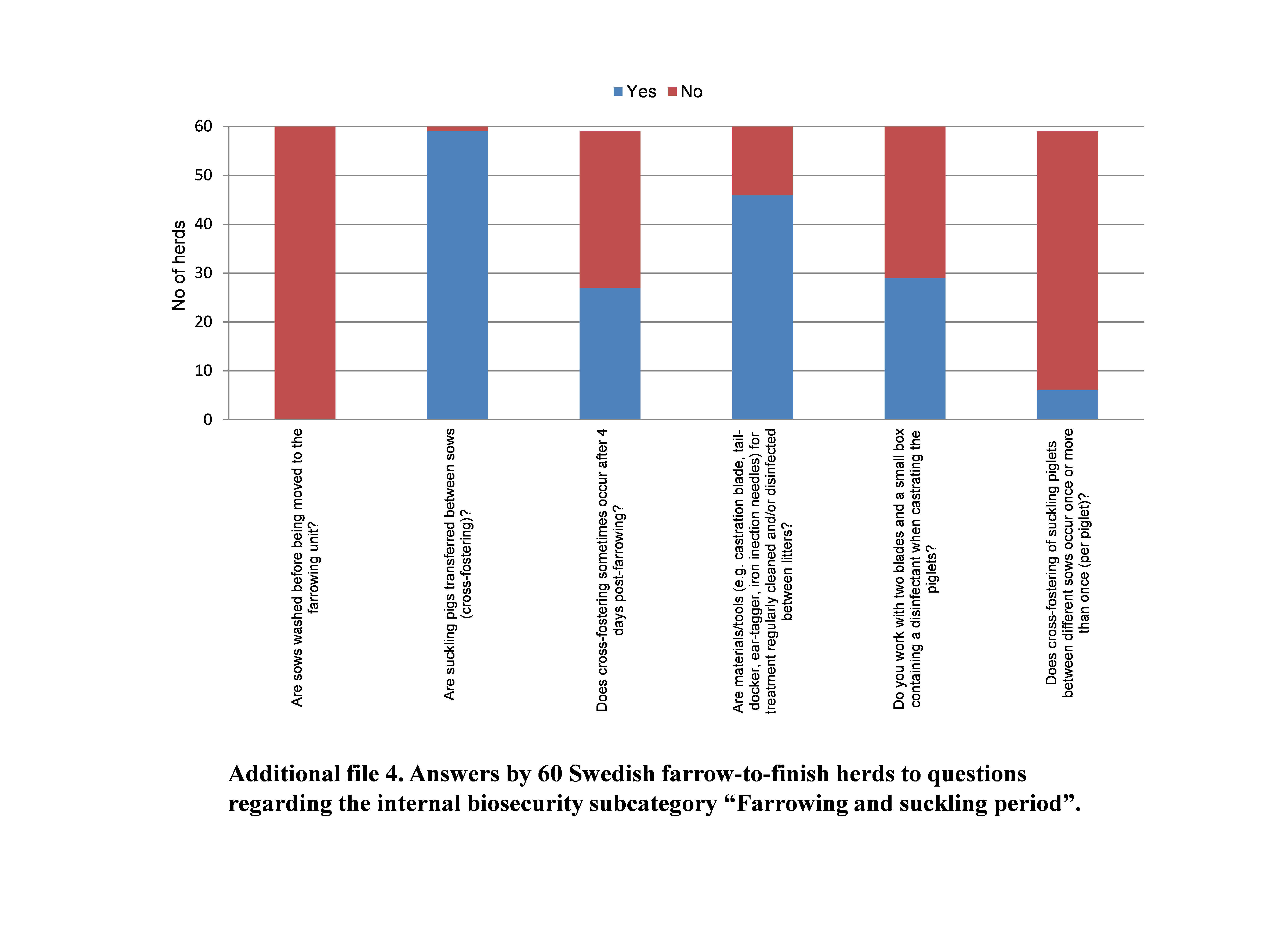

Supplement: Additional file 4: — Answers by 60 Swedish farrow-to-finish herds to questions regarding the internal biosecurity subcategory “Farrowing and suckling period”. [file 13028_2015_103_MOESM4_ESM.png]

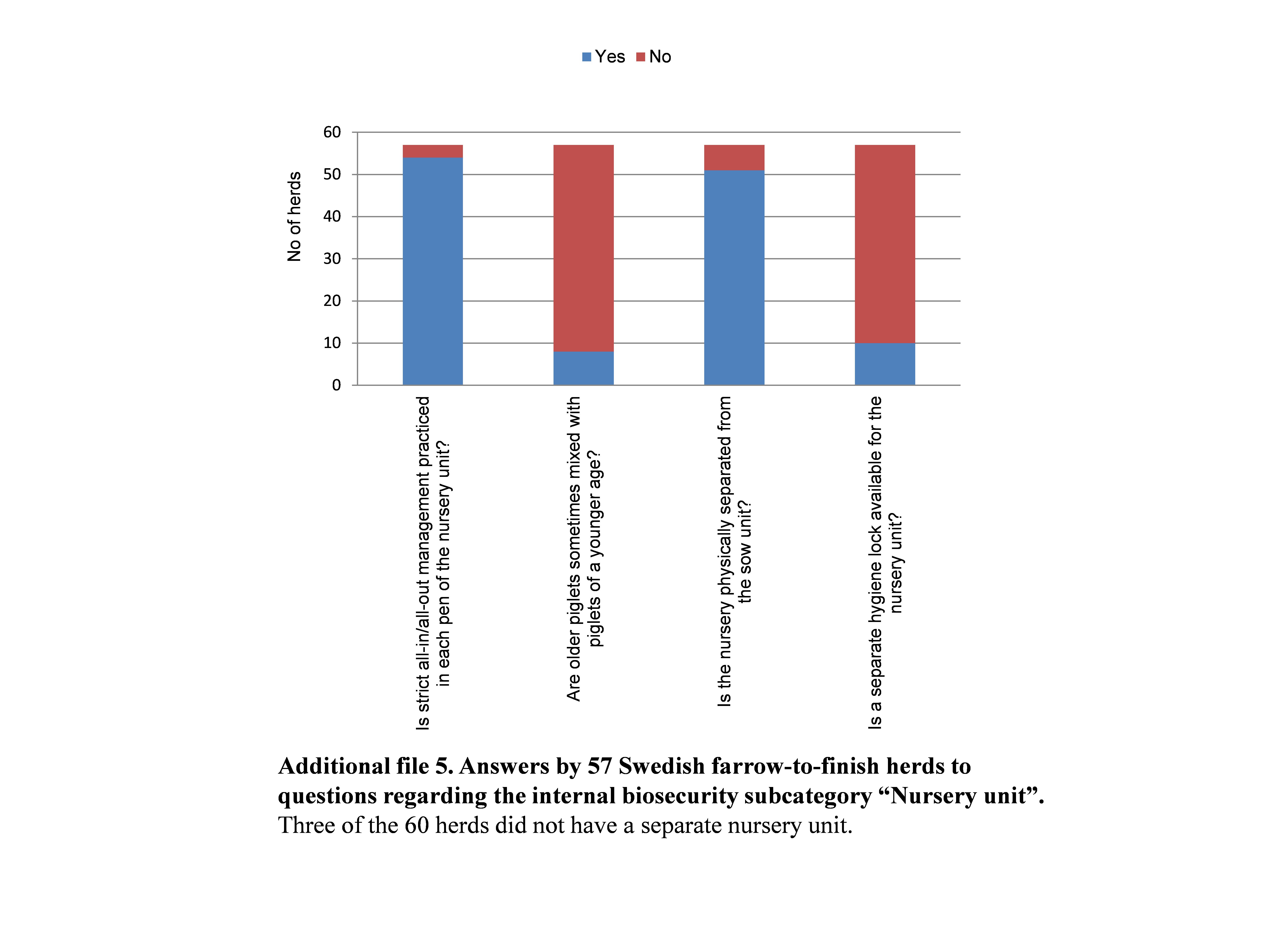

Supplement: Additional file 5: — Answers by 57 Swedish farrow-to-finish herds to questions regarding the internal biosecurity subcategory “Nursery unit”. Three of the 60 herds did not have a separate nursery unit. [file 13028_2015_103_MOESM5_ESM.png]

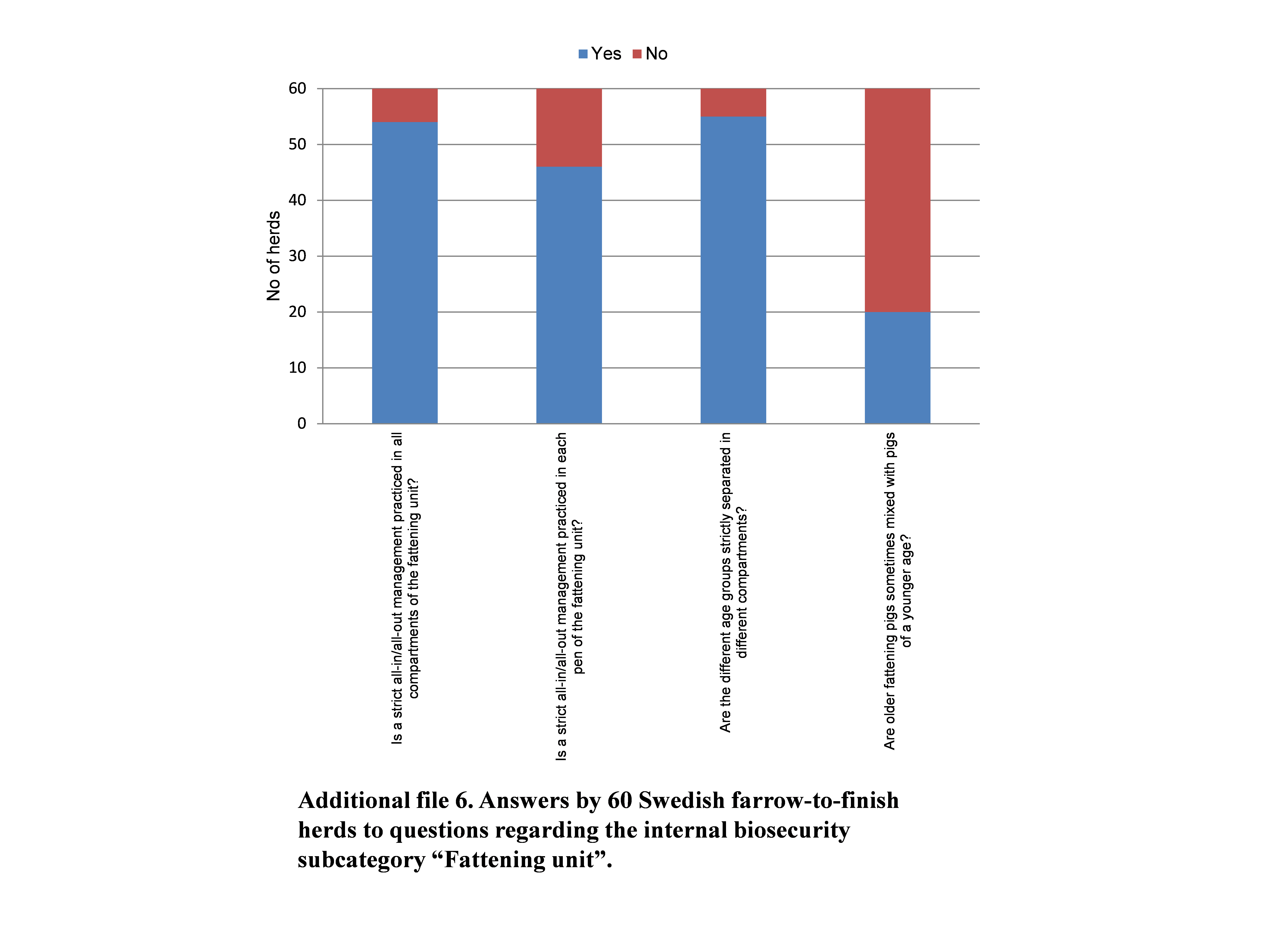

Supplement: Additional file 6: — Answers by 60 Swedish farrow-to-finish herds to questions regarding the internal biosecurity subcategory “Fattening unit”. [file 13028_2015_103_MOESM6_ESM.png]

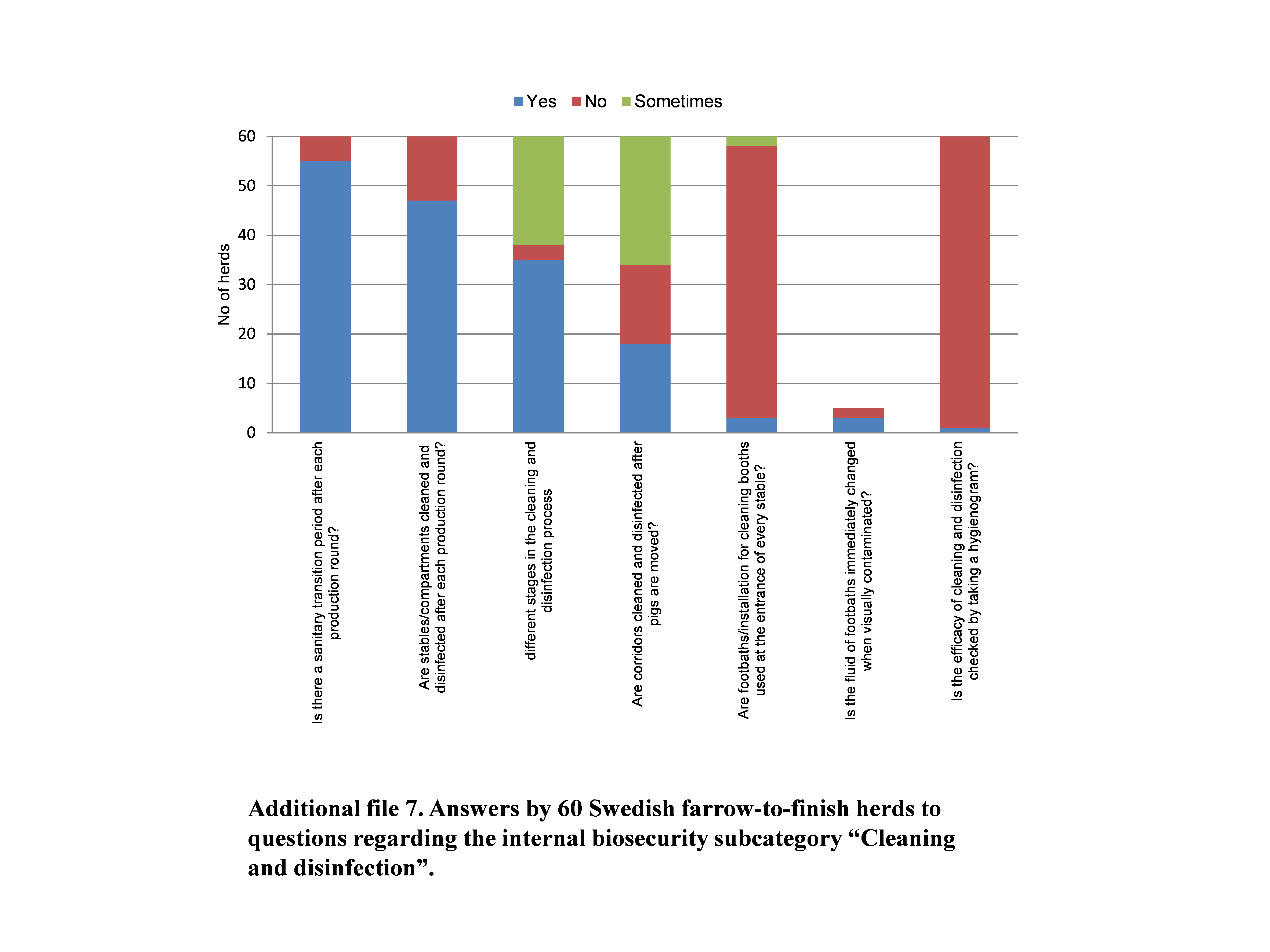

Supplement: Additional file 7: — Answers by 60 Swedish farrow-to-finish herds to questions regarding the internal biosecurity subcategory “Cleaning and disinfection”. [file 13028_2015_103_MOESM7_ESM.png]
